# Troponin Elevation Is a Potential Marker for Patients With Acute Ischemic Stroke in Intensive Care Units: A Retrospective Observational Study

**DOI:** 10.1155/srat/9914971

**Published:** 2026-03-11

**Authors:** Xianming Qiu, Lei Zhou, Bingyun Wu, Yang Yang, Brayal Dsouza, Weiwei Huang, Paulo Moreira

**Affiliations:** ^1^ Department of Critical Care Medicine, The First Affiliated Hospital of Shandong First Medical University & Shandong Provincial Qianfoshan Hospital, Shandong Medicine and Health Key Laboratory of Emergency Medicine, Shandong Institute of Anesthesia and Respiratory Critical Medicine, Jinan, Shandong Province, China; ^2^ Department of Neurology, The First Affiliated Hospital of Shandong First Medical University & Shandong Provincial Qianfoshan Hospital, Shandong Institute of Neuroimmunology, Shandong Key Laboratory of Rheumatic Disease and Translational Medicine, Jinan, Shandong Province, China; ^3^ Department of Health Care and Hospital Management, Prasanna School of Public Health, Manipal Academy of Higher Education, Manipal, India, manipal.edu; ^4^ International Healthcare Management Research and Development Center (IHM-RDC), The First Affiliated Hospital of the Shandong First Medical University and Shandong Provincial Qianfoshan Hospital, Jinan, China; ^5^ Atlantica Instituto Universitario, Gestao em Saude, Oeiras, Portugal

**Keywords:** acute ischemic stroke, cardiac troponin I, emergency medicine, inflammation, intensive care unit

## Abstract

**Objective:**

By considering risk factors of patients with acute ischemic stroke in the neurology department and intensive care unit (ICU), the study explored which indicators affected the patient’s condition, and by comparing the biochemical indicators of survival and death patients with ischemic stroke in ICU, the study explored which biomarkers can be associated with the patient’s prognosis.

**Methods:**

The study retrospectively analyzed 27 patients with acute ischemic stroke from ICU and 43 patients from the neurology department. The analysis indicators include medical history, lifestyle habits, electrocardiogram, biochemical indicators, and inflammatory cytokines. The study compared the differences in the above factors between patients admitted to the ICU and those not admitted to the ICU. Multivariable logistic regression analyses were performed to identify predictors affecting the death of patients transferred to ICU.

**Results:**

Neutrophilic granulocyte and D‐dimer in patients with ischemic stroke from ICU were significantly increased (*p* < 0.05). In ICU, compared with patients alive, we found that cardiac troponin I in patients who finally died was significantly increased (*p* < 0.05, OR is 29.250, and 95% CI is 2.789, 306.811).

**Conclusions:**

Acute ischemic stroke patients with higher neutrophils and D‐dimer were more likely to enter into the ICU. Cardiac troponin I elevation seems to be associated with the poor prognosis of patients in ICU.

**Study Highlights**

• What Is the Current Knowledge on the Topic?
  o. Evidence on understanding which biomarkers affect the patient’s prognosis is available, and it is of growing international interest, especially on patients with acute ischemic stroke (AIS).
• What Question Did This Study Address?
  o. The study explored which indicators affected the patient’s condition and, by comparing the biochemical indicators of surviving and deceased patients with ischemic stroke in ICU, the evidence generated explores which biomarkers may have affected the patient’s prognosis.
• What Does This Study Add to Our Knowledge?
  o. Our article develops an innovative approach to understanding the topic by exploring which biomarkers may affect the patient’s prognosis.
  o. The results indicate neutrophilic granulocyte (NE) and D‐dimer (D‐D) in patients with ischemic stroke from ICU were significantly increased (*p* < 0.05). In ICU, compared with surviving patients, the study suggests that cardiac troponin I (cTnI) in deceased patients was significantly increased (*p* < 0.05, OR is 29.250, and 95% CI is 2.789, 306.811).
• How Might This Contribute to Practice in Critical Care?
  o. Two main conclusions can be summarized for the international debate: (1) AIS patients with higher neutrophils and D‐D were more likely to be admitted into the ICU. (2) cTnI elevation is associated with the poor prognosis of patients in ICU.

## 1. Introduction

A version of this study was published as a preprint in Square Research [1], and it stems from an initial understanding that cerebral apoplexy (also known as stroke or cerebrovascular accident) is an acute medical disease with the main clinical manifestations of cerebral ischemia and hemorrhagic injury. AIS represents 68% of all strokes and is associated with permanent brain injury secondary to disruption of blood flow [2, 3]. Ischemic stroke, while remaining a major cause of disability and the second cause of deaths throughout the world, is identified as needing improvements in prevention as a priority for related health management development [4, 5]. Due to the limited predictive characteristics of existing models, the prognosis of ischemic stroke patients remains challenging. Blood‐based biomarkers may provide additional information for identified prognostic factors. However, there are few studies on this among the Chinese population. Thus, advancements on early and rapid diagnosis, prognosis, and optimal risk stratification, as well as secondary prevention, are required.

During AIS, blood biomarkers can be any quantifiable entity that reflects the manifestations of stroke‐related processes. Evidence suggests that multiple blood indicators are associated with stroke [6, 7]. The elevation of cTnI has been suggested to indicate the disruption of integrity of myocardial cell membrane and injury to myocardial cells. However, the role of cTnI in the prognosis of patients with AIS is still controversial. Some studies have shown that cTnI levels are elevated in AIS [8–10], indicating a close relationship between stroke and cardiac damage. Yet, evidence on the intensity of risk estimation varies greatly among different studies [11, 12]. Internationally, there are many other blood indicators being studied. One study suggests that, in patients with an ischemic stroke, estimated GFR based on serum cystatin was a better predictor of all‐cause and cardiovascular mortality [13]. A systematic review summarized blood indicators related to acute stroke from 2007 to 2018 [6] and suggested that natriuretic peptide and markers of inflammation, atherosclerosis, and stress are the most promising prognostic biomarkers in the identified studies. Increased plasma inflammatory cytokine levels and leukocytes have also been reported in ischemic stroke [14, 15]. Prospective research suggests that low levels of lncRNA NKILA may predict an increased risk of lacunar stroke [16].

Based on previous research and evidence, this study explored the serum levels of biochemical indicators, inflammatory cytokines, and cardiac risk factors to further reveal the biomarkers in patients with AIS in or out of the ICU setting. At the same time, the study also investigated the biomarkers related to the prognosis of patients with AIS in the ICU by using multivariable logistic regression analysis.

## 2. Materials and Methods

### 2.1. Study Population

The consecutive patients who were admitted to the hospital due to AIS from May 2020 to December 2021 were retrospectively evaluated. Twenty‐seven patients with AIS (15 men, mean age 75 years) from ICU and 43 patients with AIS (26 men, mean age 74 years) from neurology were enrolled. The study protocol was approved by the Hospital ethics committee.

Because the study at present was a retrospective design, the written informed consent was waived. The following inclusion criteria were used for the patients with AIS: fulfilled the World Health Organization definition criteria for the sudden neurological deficit that has a presumable vascular etiology. Brain computed tomography (CT) scan was normal, or diffusion‐weighted imaging magnetic resonance imaging (MRI) showed acute ischemic changes. At admission, measurement of biochemical indicators, inflammatory cytokines, and ECG were routinely performed in all patients. The following inclusion criteria were used for all patients: evidence of acute coronary syndrome, impaired renal function (estimated glomerular filtration rate < 60 mL/min per 1.73 m^2^), congestive heart failure (having a history of heart failure or reduced ejection fraction ≤ 40*%*), and hepatic and endocrinal diseases as assessed by clinical history, physical examination, or routine laboratory test.

### 2.2. Measurement of Plasma Biochemical Indicators

Peripheral blood was drawn from patients with AIS, and white blood cells (WBCs), NE, platelets (PLTs), hemoglobin (Hb), hematocrit (HCT), homocysteine (HCY), alanine aminotransferase (ALT), aspartate aminotransferase (AST), albumin (Alb), blood urea nitrogen (BUN), creatinine (CRE), uric acid (UA), D‐D, triglyceride (TAG), total cholesterol (TC), high‐density lipoprotein (HDL), and low‐density lipoprotein (LDL‐C) content were extracted and measured in the laboratory.

### 2.3. Cardiac Investigation

cTnI was measured as a part of routine laboratory testing on admission. The significant elevation of the level of troponin I was considered if it was ≥ 0.24 ng/mL. All patients underwent 12‐lead ECG at admission, and the data were analyzed by cardiologists according to a modified version of the Minnesota code. Corrected QT intervals (QTc) were defined as prolonged if the QTc in lead II was ≥ 460 ms in women and ≥ 450 ms in men.

### 2.4. Statistical Analysis

Categorical variables were compared with the chi‐square test for the difference in rates between different groups (comparison between patients admitted to the ICU and those not admitted to the ICU); for numerical variables, the normality test was performed first, and the test was used for the two groups if they were in line with the normal distribution. For the comparison of the mean values, the nonparametric rank sum test was used to compare the differences between the two groups for the numerical variables that did not conform to the normal distribution. Incorporate the abovementioned meaningful variables into the backward stepwise unconditional logistics regression model to analyze the factors affecting whether to stay in the ICU. Multivariable logistic regression analyses were performed to identify predictors affecting the death of patients transferred to ICU. *p* < 0.05 was considered to be significant. All statistical analyses were performed using SPSS 17.0 for Windows (SPSS Inc, Chicago, IL).

## 3. Results

Demographic characteristics and medical history in patients with AIS from ICU and neurology were shown in Table 1. A total of 27 patients with AIS from ICU and 43 patients with AIS from neurology were recruited for the present clinical study. The mean age was 75 and 74 years in cerebral apoplexy patients from ICU and neurology, respectively. The numbers of males and females in the two groups were approximately equal. There were no statistical differences in medical history (hypertension/diabetes/atrial fibrillation/chronic kidney disease), life habits (smoking/drinking), or ECG changes of as‐included patients.

**Table 1:** Comparison of demographic characteristics and medical history in patients with AIS.

	Total (*n* = 70)	Admission to ICU (*n* = 27)	No admission to ICU (*n* = 43)	*p* value
Age, years		75 (67, 76)	74 (67, 80)	0.629
Male gender	41	15	26	0.437
Smoke	24	9 (60%)	15 (57%)	0.552
Alcohol	25	11 (26%)	14 (52%)	0.329
Hypertension	52	18 (%)	34 (%)	0.190
Diabetes mellitus	30	9 (%)	21 (%)	0.152
Atrial fibrillation	22	8 (%)	14 (%)	0.506
Chronic kidney disease	3	0 (%)	3 (0)	0.225
ST‐T abnormal	36	16 (%)	20 (%)	0.214
*T* wave abnormal	9	4 (%)	5 (%)	0.483
*Q* wave abnormal	12	5 (%)	7 (%)	0.526

*Note:* Data are presented as median (interquartile range) or *n* (%) unless other indicated. *p* < 0.05 indicated statistically significant difference.

There was no statistically significant difference in Hb, HCT, PLT, HCY, ALT, AST, Alb, BUN, CRE, UA, TAG, TC, HDL, or LDL, whereas WBC, NE, and D‐D in patients with AIS from ICU were significantly increased when compared with patients from neurology (*p* < 0.05, Table 2).

**Table 2:** Comparison of laboratory examination results in patients of AIS.

	Admission to ICU (*n* = 27)	No admission to ICU (*n* = 43)	*p* value
WBC, 10^9^/L	10.61 (8.64, 12.865)	8.12 (6.53, 9.49)	0.001
NE, %	81.46 ± 8.64	70.04 ± 12.26	0.01
Hb, g/L	133 (115.5, 145)	135 (119.5, 148)	0.433
HCT, %	40.71 ± 6.68	41.26 ± 6.72	0.741
PLT, 10^9^/L	251 (161.5, 283)	215 (167, 256.5)	0.507
HCY,	14.3 (11.6, 23.1)	15.15 (12.58,19.03)	0.801
ALT, U/L	13.5 (11.9, 23.1)	21.05 (13.35, 31.35)	0.293
AST, U/L	27.2 (18.25, 42.32)	21.85 (17.78, 32.35)	0.296
Alb, g/L	37.34 ± 5.57	38.94 ± 4.49	0.198
BUN, mmol/L	6.3 (4.75, 7.95)	5.4 (4.5, 7)	0.319
CRE, *μ*mol/L	78 (61.75, 93.55)	69 (58.9, 86.5)	0.546
UA, *μ*mol/L	269 (222.8, 319.75)	314 (235.8, 360.7)	0.165
TAG, mmol/L	0.94 (0.74, 1.235)	1.02 (0.87, 1.66)	0.102
TC, mmol/L	4.27 ± 1.26	4.15 ± 1.38	0.692
HDL, mmol/L	1.19 (0.875, 1.275)	1.04 (0.83, 1.31)	0.344
LDL, mmol/L	2.53 ± 0.91	2.33 ± 0.89	0.374
D‐D, ng/mL	7.17 (1.33, 9.57)	0.82 (0.43, 1.46)	0.001
TnI, ng/mL	0.37 ± 0.48	0.58 ± 0.49	0.079

*Note:* Data are presented as mean ± SD or median (interquartile range) or *n* (%). *p* < 0.05 indicated statistically significant difference.

Abbreviations: Alb, albumin; ALT, alanine aminotransferase; AST, aspartate aminotransferase; BUN, blood urea nitrogen; CRE, creatinine; D‐D, D dimer; Hb, hemoglobin; HCT, hematocrit; HCY, homocysteine; HDL, high‐density lipoprotein; LDL‐C, low‐density lipoprotein; NE, neutrophilic granulocyte; PLT, platelets; TAG, triglyceride; TC, total cholesterol; UA, uric acid; WBC, white blood cell.

The three variables of WBC, NE, and D‐D were included in stepwise backward logistic regression. In Step 1, all three variables were included, and the *p* value (Sig.) of WBC was the least significant, which was removed in Step 2. In Step 2, the NE and D‐D showed significant results (*p* < 0.05, Table 3). The increase of neutrophil percentage and D‐D was predictive for worse prognosis in patients with AIS, which means they were more likely to be admitted to the ICU.

**Table 3:** Logistic regression analysis of prognosis in patients with AIS.

	B	OR	95% CI	*p* value
NE, %	7.891	2673.101	1.005, 3.189	0.032
D‐D, ng/mL	0.434	1.544	1.013–1.150	0.002

When the condition of patients with AIS continues to deteriorate, they need to get into ICU for further treatment. A total of 13 patients with AIS from ICU were dead and 14 patients got better. There was no statistically significant difference in their age, gender, medical history (hypertension/diabetes/atrial fibrillation), life habits (smoking/drinking), ECG changes, Hb, HCT, PLT, HCY, ALT, AST, Alb, BUN, CRE, UA, TAG, TC, HDL, LDL, WBC, NE, or D‐D, whereas cTnI in patients who finally died was significantly increased when compared with patients who are alive (*p* < 0.05, Tables 4 and 5). cTnI was divided into high and low groups to analyze the impact of cTnI on the death of patients with AIS in ICU, and we found that elevated cTnI is a risk factor for death in patients with AIS (*p* < 0.05, OR is 29.250, and 95% CI is 2.789, 306.811, as shown in Table 6).

**Table 4:** Comparison of DEMOGRAPHIC characteristics and medical history in dead and alive patients in ICU.

	Total (*n* = 27)	Dead (*n* = 14)	Alive (*n* = 13)	*p* value
Male gender	15	7	8	0.704
Smoke	9	4	5	0.695
Alcohol	11	6	5	1.000
Hypertension	18	9	9	0.555
Diabetes mellitus	9	4	5	0.695
Coronary heart disease	5	3	2	0.538
Atrial fibrillation	8	4	4	0.615
ST‐T abnormal	16	8	8	0.564
*T* wave abnormal	4	2	2	0.673
*Q* wave abnormal	5	2	3	0.648

*Note:* Data are presented as *n* (%) unless other indicated. *p* < 0.05 indicated statistically significant difference.

**Table 5:** Comparison of laboratory examination results in dead and alive patients in ICU.

	Dead (*n* = 14)	Alive (*n* = 13)	*p* value
Age, years	75.5 (66.0, 76.0)	72.0 (67.5, 78.5)	0.595
TnI, ng/mL	0.78 (0.053, 4.33)	0.025 (0.017, 0.22)	0.005 ^∗^
WBC, ^∗^10^9^/L	10.45 (8.67, 15.29)	10.61 (8.46, 12.02)	0.356
NE, %	86.0 (73.0, 91.8)	80.9 (75.35, 86.55)	0.286
Hb, g/L	135.43 ± 28.96	129.08 ± 19.79	0.515
HCT, %	41.57 ± 7.98	39.78 ± 5.44	0.505
PLT, ^∗^10^9^/L	262.5 (160.2, 322.5)	249.0 (152.0, 278.5)	0.544
HCY,	17.6 (11.6, 36.9)	12.5 (10.75, 17.60)	0.109
ALT, U/L	13.5 (12.12, 33.17)	14.0 (11.2, 32.45)	0.942
AST, U/L	28.4 (19.07, 42.83)	25.0 (15.95, 42.9)	0.467
Alb, g/L	37.21 ± 6.03	37.49 ± 5.53	0.904
BUN, mmol/L	7.0 (4.78, 7.87)	5.9 (4.0, 9.15)	0.808
CRE, *μ*mol/L	76.9 (66.05, 100.87)	78 (54.25, 88.35)	0.207
UA, *μ*mol/L	276.3 (221.2, 334.1)	263 (205.8, 319.75)	0.560
TAG, mmol/L	0.97 (0.81, 1.50)	0.83 (0.57, 1.27)	0.254
TC, mmol/L	4.39 ± 1.36	4.15 ± 1.24	0.624
HDL, mmol/L	0.98 (0.87, 1.24)	1.24 (0.91, 1.53)	0.198
LDL, mmol/L	2.70 ± 0.96	2.35 ± 0.89	0.341
D‐D, ng/mL	8.11 (4.99, 15.12)	2.38 (0.78, 8.61)	0.152

^∗^ *p* <0.05.

**Table 6:** Logistic regression analysis of cTnI.

	B	OR	95% CI	*p* value
cTnI	3.376	29.250	2.789, 306.811	0.005 ^∗^

^∗^ *p* <0.05.

## 4. Discussion

Stroke diagnosis could be challenging in the acute phase. Many traditional and new blood markers are used to make appointments about the prognosis of stroke patients [17, 18]. However, the studied biomarkers were not sufficient for predicting the prognosis of stroke. In our study, acute stroke patients with higher neutrophils and D‐D were more likely to enter the ICU. It can be seen that inflammation and thrombosis were the main reasons for taking charge.

Neutrophils are one of the first cells to respond during ischemic stroke. Neutrophils have been reported to be associated with the morbidity and severity of cerebral apoplexy [19]. The mechanism may be related to the following two aspects: (1) Neutrophils accumulate in the blood vessels, blocking capillary blood flow, aggravating post‐ischemic microvascular injury, and hindering blood flow reperfusion [20]. (2) Neutrophils are an important component of acute inflammation in ischemic stroke, and neutrophils are activated by DAMPs‐induced toll‐like receptors (TLRs) of resident cells, releasing leukotrienes, cytokines, chemokines, ROS, and various proteases such as MPO, NE, and MMP‐9, which directly damage the endothelium, resulting in increased permeability of the blood–brain barrier, edema of brain tissue, further blocking blood vessels, and releasing NETs. This, in turn, promotes tissue and blood–brain barrier damage [20, 21]. At the same time, neutrophils can participate in the formation and expansion of thrombosis, which in turn impairs blood circulation remodeling and vascular remodeling after stroke and exacerbates ischemic neuronal injury [20]. D‐D is a marker of haemostatic imbalance. Many studies have shown that elevated levels of D‐D are an independent risk factor for poor prognosis in stroke [22]. The reason may be the release of coagulation factors and increased intracranial pressure from brain tissue damage after cerebral infarction, leading to an imbalance of high coagulation and low fibrinolysis in the body [23].

Stroke patients entering into the ICU will receive active treatment; nevertheless, some patients get better while others die. So what factors are related to the prognosis of patients? We retrospectively analyzed the factors that affect the prognosis of stroke patients in the ICU. Our research shows that patients with elevated troponin had a worse prognosis. With stroke and IHD sharing the same risk factors, it is not unexpected to see the two diseases in one patient. It is not surprising to see both stroke and IHD in a patient as they share the same risk factors. Many studies have shown a high percentage of troponin in patients with acute stroke [24]. A possible explanation for elevated troponin levels in patients with AIS may be a coincidence of acute coronary syndromes leading to the elevated circulating troponin levels [25]. The susceptibility of the heart to neurogenic stress may be another explanation for the elevated troponin [26, 27]. Activation of the sympathetic adrenal system may be an important factor for myocardial injury in patients with AIS [1]. Another meta‐analysis showed that the hospital and nonhospital mortality rates of AIS patients with elevated cTnT or cTnI were increased [4]. However, the etiology of elevated troponin in acute stroke is not entirely clear. It is speculated that damage to the centers regulating autonomic nervous function could lead to autonomic dysfunction, leading to sympathetic overflow and neurogenic myocardial injury [1].

In summary, acute stroke patients with higher neutrophils and D‐D were more likely to enter into the ICU, and the elevation of cTnI is associated with the poor prognosis of patients in the ICU. Patients with inflammation, thrombosis, and elevated cTnI levels should be closely monitored and receive appropriate care to improve their conditions.

## 5. Limitations

We did not measure the size of the infarct, and the determination of the results was not part of our research design. Some previous studies found no correlation between stroke location and troponin levels [1]. The limitations of our study mainly include a small sample size, single‐center design, and lack of long‐term follow‐up. The findings should be interpreted with caution, given the exploratory nature of this study. Future research with larger cohorts is needed to validate these results.

## Author Contributions

The authorship requirements have been met. Xianming Qiu developed the project and wrote the original manuscript. Lei Zhou undertook the data validity. Yang Yang interpreted the statistical results. Bingyun Wu interpreted the results and edited the manuscript. Weiwei Huang provided study supervision and contributed to the study concept. Brayal Dsouza revised the data and statistics accuracy. Paulo Moreira revised the whole manuscript, quality of data, and interpretation and discussion.

## Funding

This work was supported by the Shandong Medical and Health Science and Technology Development Project (2017WS189) and research support from the Chinese Sleep Research Society Hansoh Project (2019HB02).

## Disclosure

Part of the manuscript was published as a preprint in Square Research (10.21203/rs.3.rs-3343991/v1). The final manuscript was approved by all authors.

## Ethics Statement

The study protocol was approved by the Ethics Committee of the Shandong Provincial Qianfoshan Hospital (XMSBLL2023 (082)) and was in accordance with the principles described in the Declaration of Helsinki. The study protocol was described to each subject, and written informed consents were obtained.

## Conflicts of Interest

The authors declare no conflicts of interest.

## Data Availability

The data that support the findings of this study are available from the corresponding authors upon reasonable request.
